# Meta-analysis of the effects of proton pump inhibitors on the human gut microbiota

**DOI:** 10.1186/s12866-023-02895-w

**Published:** 2023-06-19

**Authors:** Jiayi Zhang, Chengcheng Zhang, Qingsong Zhang, Leilei Yu, Wei Chen, Yuzheng Xue, Qixiao Zhai

**Affiliations:** 1 State Key Laboratory of Food Science and Technology, Jiangnan University, Wuxi, Jiangsu 214122 People’s Republic of China; 2 School of Food Science and Technology, Jiangnan University, Wuxi, 214122 Jiangsu China; 3 Department of Gastroenterology, Affiliated Hospital of Jiangnan University, Jiangsu Province Wuxi, China

**Keywords:** Gut microbiota, Proton pump inhibitors, Meta-analysis, Supervised learning technique, Biomarkers

## Abstract

Mounting evidence has linked changes in human gut microbiota to proton pump inhibitor (PPI) use. Accordingly, multiple studies have analyzed the gut microbiomes of PPI users, but PPI–microbe interactions are still understudied. Here, we performed a meta-analysis of four studies with available 16S rRNA gene amplicon sequencing data to uncover the potential changes in human gut microbes among PPI users. Despite some differences, we found common features of the PPI-specific microbiota, including a decrease in the Shannon diversity index and the depletion of bacteria from the Ruminococcaceae and Lachnospiraceae families, which are crucial short-chain fatty acid-producers. Through training based on multiple studies, using a random forest classification model, we further verified the representativeness of the six screened gut microbial genera and 20 functional genes as PPI-related biomarkers, with AUC values of 0.748 and 0.879, respectively. Functional analysis of the PPI-associated 16S rRNA microbiome revealed enriched carbohydrate- and energy-associated genes, mostly encoding fructose-1,6-bisphosphatase and pyruvate dehydrogenase, among others. In this study, we have demonstrated alterations in bacterial abundance and functional metabolic potential related to PPI use, as a basis for future studies on PPI-induced adverse effects.

**Supplementary Information:**

The online version contains supplementary material available at 10.1186/s12866-023-02895-w.

## Introduction

Many studies are being conducted recently on the interaction between drug use and the gut microbial ecosystem. Exploring the associations between commonly used drugs, such as antibiotics and metformin, and the gut microbe composition are conducive to understanding mechanisms underlying the effects of drugs including their potential side effects [1, 2]. Proton pump inhibitors (PPIs), which have long been used globally [3, 4], comprise one of the most common types of prescription drugs, known to efficiently inhibit gastric acid secretion. The clinical efficacy of PPIs, including omeprazole, lansoprazole, and pantoprazole, have been established against many diseases, including peptic ulcer disease, gastroesophageal reflux, non-steroidal-induced gastrointestinal lesions, Helicobacter pylori infection, and eosinophilic esophagitis [5]. These drugs covalently bind to H + /K + -ATPase antiporter pumps of gastric parietal cells, preventing hydrogen ions from being released into the stomach cavity and increasing the gastric ph [6]. The effects of PPIs on the gut microbiome reportedly can be explained by a combination of two mechanisms, specifically the direct inhibition of some commensal gut bacteria, such as Ruminococcus and Dorea species and the indirect stimulation of the growth of some typical oral bacteria, which is mediated by the increase in the gastrointestinal pH [7–9].

However, long-term PPI use impacts the survival and induces migration of multiple bacteria along the gastrointestinal tract [10], consequently increasing the risk of gut dysbiosis [9, 11, 12]. Imhann et al. (2016) studied the gut microbiome composition of 1815 volunteers, and the results showed that 20% of bacterial taxa including the genera *Rothia*, *Enterococcus*, *Streptococcus*, and *Staphylococcus* were changed predisposing the individual to *Clostridium difficile* infections (CDI). Llorente et al. (2017) also found that PPI-induced overgrowth of *Enterococcus* will in turn exacerbate ethanol-induced liver disease in mice and humans [13]. Furthermore, the overutilization of PPIs can cause bacterial overgrowth in the small intestine and increase the risk of enteric infections, such as those caused by *Campylobacter*, *Shigella*, and *Salmonella* [14–16]. A systematic review incorporating 12 observational cohorts and 11 interventional cohorts revealed that PPIs can change the microbiota of the upper and end of the intestine to some extent (e.g., Pasteurellaceae, Enterobacteriaceae, Ruminococcaceae, and Lachnospiraceae) [17].

Gut dysbiosis [18] caused by PPIs is closely related to multiple adverse effects including vitamin and mineral deficiency, fractures and osteoporosis, chronic liver diseases, and other extraintestinal complications [19–21], which will affect the health of the users to some extent. PPI use may promote infections in patients with decompensated liver cirrhosis due to chronic Hepatitis C Virus infection, either directly or indirectly through changes in the microbial community structure [22]. However, a meta-analysis focusing on the associations between PPIs and the human gut microbiome has not been performed to date. This research presents a meta-analysis based on four clinical studies. Through univariate analysis and a supervised classification model, we analyzed the gut microbiota changes related to PPI use and pinpointed some common characteristics of altered bacterial taxa and functional pathways.

## Methods

### Study selection and data acquisition

Google Scholar was searched for publications that contained all the words “gut”, “PPI”, the exact phrase “proton pump inhibitor”, at least one of the words “microbiota” [OR] “microbiome” [OR] “gut” [OR] “intestinal” anywhere in the article. As on the 12th of July 2022, 10,700 entries were obtained after the search. Titles and abstracts were then manually screened and if they contained the words “microbiome” or “microbiota” and “proton pump inhibitor” the paper was further checked. At last, a number of 158 papers were accepted for the next step screening.

For a study to be included in the meta-analysis, we accepted any type of cohorts from any countries, any method to acquire and analyze samples, and any type of study design, including cohort studies, case control studies, or cross-sectional studies [23]. We eliminated all unavailable studies including the cases in which the raw reads were either from animals or were restricted. Therefore, we considered 21 papers (Table S1) and only four studies, all using 16S rRNA gene sequencing, were finally included in our meta-analysis. The country codes used for the different studies were AT1, AT2, CN, US (Table 1). Except for one study in which metadata were provided by the author [11], sequencing data of the other three studies were retrieved from the NCBI short read archive (SRA). The SRA identifiers were: SRP119055 by Castellani et al. [24], PRJNA648014 by Lin et al. [25], and SRP132827 for Horvath et al. [26].

**Table 1:** Technical information of the four incorporated studies

Reference	Country code	Design	No. of cases	No. of controls	Medication	DNA extraction	16S Region	Sequencing platform
Horvath et al.	Austria 1	Case–control	62	64	NA	MagNA Pure LCDNA Isolation Kit	v1–v2	MiSeq
Castellani et al.	Austria 2	Self-control	12	/	8 weeks	PSP-Spin Stool DNA Kit	v1–v2	MiSeq
Lin et al.	China	Case–control	58	60	At least 1 month	QIAamp DNA Stool Mini Kit	v3–v4	MiSeq
Freedberg et al.	USA	Self-control	12	/	8 weeks	PowerFecal DNA Isolation Kit	v4	MiSeq

NA means relevant information was not reported in the included studies

### Data reprocessing and reanalysis

Sequencing data from each selected study were reprocessed separately [23]. We use QIIME2 [27] and the Silva database [28] to perform sequence quality control and build the ASV (amplicon sequence variant) feature table [29]. Specifically, to remove low-quality sequences and obtain representative sequences, raw reads were filtered based on quality using the QIIME2 DADA2 plugin. Owing to the different sequencing regions associated with by different studies, the selected representative sequences were next classified and annotated using the trained Silva database feature classifier (https://data.qiime2.org/2020.8/common/silva-138-99-nb-classifier.qza). To this end, the ASV feature tables were downloaded from the QIIME2 viewer (https://view.qiime2.cn/visualization/) and converted into relative abundance data for subsequent diversity analysis. The samples were grouped into PPI and CTRL groups to perform further downstream analyses. Among them, samples from healthy controls and non-PPI patients were defined as the CTRL group, and samples from PPI patients were defined as the PPI group. The baseline samples were regarded as the CTRL group, whereas the last samples provided after medication administration were regarded as the PPI group in the two self-control studies.

### Composition and diversity analysis

Taxonomic relative abundances at the genus level were then used to compute microbial diversities and dissimilarities. Bray–Curtis distance and principal coordinates analysis were performed using the functions *vegdist* (*method* = *“bray”*) and *cmdscale* in the R package *vegan*. The Shannon index was calculated using the function *diversity* (*index* = *“shannon”*). Following the protocol of Ho et al. [30], we employed the fixed effect meta-analysis model to pool the adjusted estimates and standard errors of diversity indexes from all included studies via inverse variance weighting of the between-study variances. For the fixed effects model, we referred to Zhang et al. [31].

### Differential abundance analysis

To identify the differentially present bacteria, differential abundance analysis was carried out. We first chose 77 genera with a relative abundance greater than 0.1%, all of which were highly detectable in the included studies. The significance of differential abundance in all four studies separately and in the meta-analysis was tested using the Wilcoxon test function in R. Significance levels were then corrected using the Benjamini–Hochberg method [32]. Furthermore, generalized fold-change values were used to summarize the significance of differentially abundant genera, aiming to provide better resolution for sparse microbiome maps. The generalized fold-change for each sample was calculated by taking the logarithm of the *P*-values of all samples of each included study and dividing it by the median value [33].

### Functional properties predicted using PICRUSt

Function prediction of 16S rRNA amplicon data were further investigated in the three studies with source data. The study with the country code “US” [11] was excluded from the functional analysis owing to the lack of an input file. Using the ASV feature table as an input file, functional compositions of gut microbiomes based on 16S rRNA sequencing data were inferred using Phylogenetic Investigation of Communities by Reconstruction of Unobserved States (PICRUSt) (https://github.com/picrust/picrust2) [34] and Kyoto Encyclopedia of Genes and Genomes (KEGG) database (https://www.genome.jp/kegg/ko.html). The feature table of 16S rRNA sequencing data was first converted into a BIOM file for processing using PICRUSt2, along with representative sequences. Then, the scripts picrust2_pipeline.py and add_descriptions.py were used to add annotations. Finally, the values for the relative abundance of functional genes were obtained. Differential abundance analysis of functional genes was performed analogous to the steps of differential genera identification (see 2.4). KEGG pathway enrichment analysis was then conducted utilizing the screened differential genes. Using the Omicshare tool (https://www.omicshare.com/tools/home/report/koenrich.html), the KEGG metabolic pathways were retrieved from the KO (KEGG ORTHOLOGY) Database [35–37] (https://www.genome.jp/kegg/ko.html), which were mapped with KOs.

### Random forest classifiers

A random forest classification model was used to identify biomarkers in relation to the gut microbiota after PPI use. Samples were randomly separated into a testing and training set. Eighty percent of the data were grouped as the training dataset to train the random forest model, and 20% were used as the test dataset to validate PPI use in research subjects. The *randomForest* package in R was used to build the random forest model, the parameters of which were then tuned using the *confusionMatrix* function in the R package *caret* [38]. Based on the receiver operating characteristic (ROC) curve and the area under the ROC curve (AUC) (*pROC* package), the accuracy of the model with respect to its ability to classify samples of the test and validation set was evaluated [39]. To identify the most discriminatory samples between the PPI and CTRL groups, predictor variables were determined based on the ranked MeanDecreaseAccuracy. Finally, differential genera and functional genes were screened from the predictor variables based on differential *P*-values between the PPI and CTRL group.

### Statistical analysis

All Wilcoxon tests were performed using the Wilcoxon test function in R. *P*-values < 0.05 and false discovery rate (FDR)-adjusted *P*-values < 0.1 were regarded as significant. All analyses and plots were performed using R statistical software version (4.2.1) and GraphPad Prism8.

## Results

### Consistent data processing of the meta-analysis

For our meta-analysis, four studies that were conducted using 16S rRNA gene sequencing were utilized to examine the influences of PPI use on the human gut microbiome. These four studies showed obvious imparities in medication, DNA extraction protocols, and the sequencing region (Table 1, Table S1). Moreover, baseline characteristics of study subjects exists differences (Table S2). To rule out heterogeneity factors during the bioinformatic analysis [33], all raw reads were reprocessed through QIIME2 for bacterial taxonomic profiling [27] and PICRUSt2 for functional profiling [40].

### Microbial diversity and PPI use

**Fig. 1:**
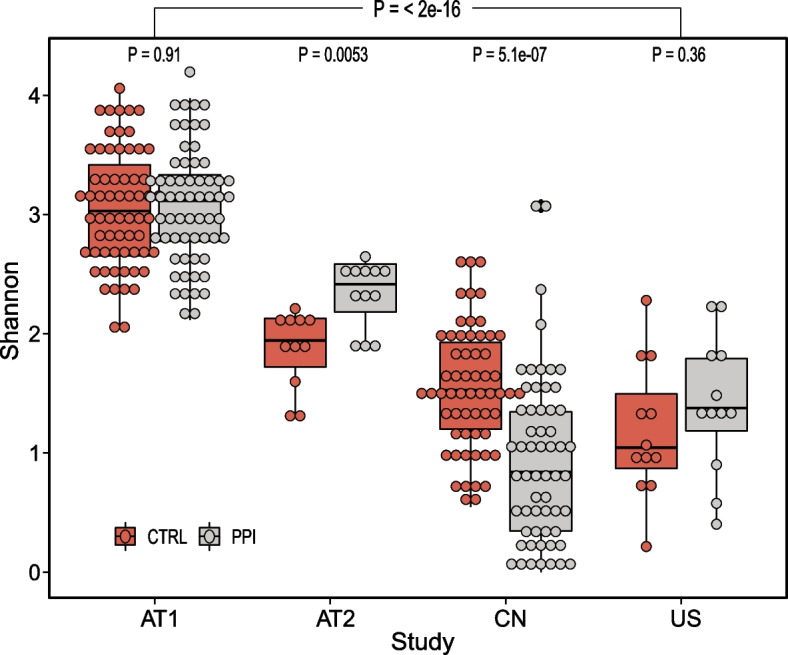
Alpha diversity index demonstrates inconsistent effects of proton pump inhibitor (PPI) use on the gut microbiota among studies

**Fig. 2:**
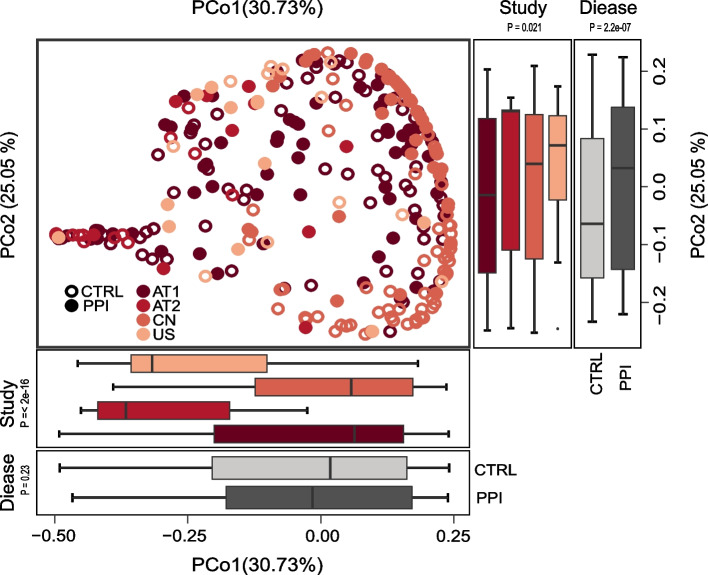
Beta diversity values of the gut microbiota differs between different studies

We first investigated the influences of PPI use on gut microbial diversity. In total, 315 taxonomical end points from 292 total stool samples from three countries were evaluated in the meta-analysis. As described in Fig. 1, PPI use induced an increase in diversity in three studies (AT2: *P* = 0.0053, CN: *P* = 5.1e-07, US). Furthermore, there were also significant differences in the Shannon index among the four included studies (*P* = 2e-16). The principal coordinate analysis based on the Bray–Curtis distance indicated significant differences in the overall microbiome structure between the PPI and CTRL group participants, which was caused by the study and medication status (Pco1: study, *P* = 2e-16; Pco2: study, *P* = 0.021, disease, *P* = 2.2e-07; Fig. 2).

**Fig. 3:**
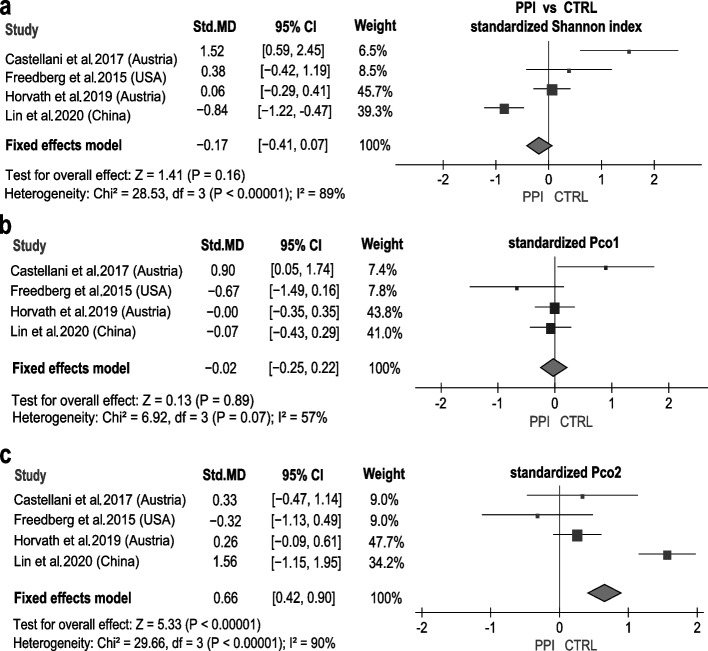
The standardized diversity indices (Shannon, PCo1, and PCo2) and results of pooled effect analysis of the four included studies

As study-associated heterogeneity has a strong influence on the microbiome composition [33], we evaluated the aforementioned results of diversity by performing a meta-analysis, by pooling the estimates from the four included studies [30]. Our results revealed a decrease in the gut microbial alpha diversity (Shannon index) among PPI users (Shannon index: pooled standardized mean difference [MD] =  − 0.17, 95% confidence interval [95% CI] = [− 0.41, 0.07], fixed effects model pooled *P*-value < 0.00001; Fig. 3a). Significant differences, as analyzed based on the fixed effects model, were also found in the gut microbiome structure between the PPI and CTRL groups (Pco1: pooled standardized MD =  − 0.02, 95% CI = [− 0.25, 0.22], fixed effects model pooled *P*-value = 0.07; Pco2: pooled standardized MD = 0.66, 95% CI = [0.42, 0.90], fixed effects model pooled *P*-value < 0.00001; Fig. 3b, c). Overall, our results showed significant alteration of gut microbial diversity related to PPI use.

The Shannon indices for all the four studies was measured. *P*-values were calculated using Wilcoxon test. AT1, AT2, CN, and US were the country codes used for four different studies.

Studies are color-coded and medication (proton pump inhibitor [PPI] versus CTRL) is distinguished by shaded/unshaded circles. The boxplots on the right and below show the projection of the 292 samples onto the first two principal coordinates, presented based on the study and medication, respectively. *P*-values were calculated using the Wilcoxon test. Country codes are provided in Fig. 1.

### Univariate meta-analysis of microbial genera and functional genes associated with PPI use

**Fig. 4:**
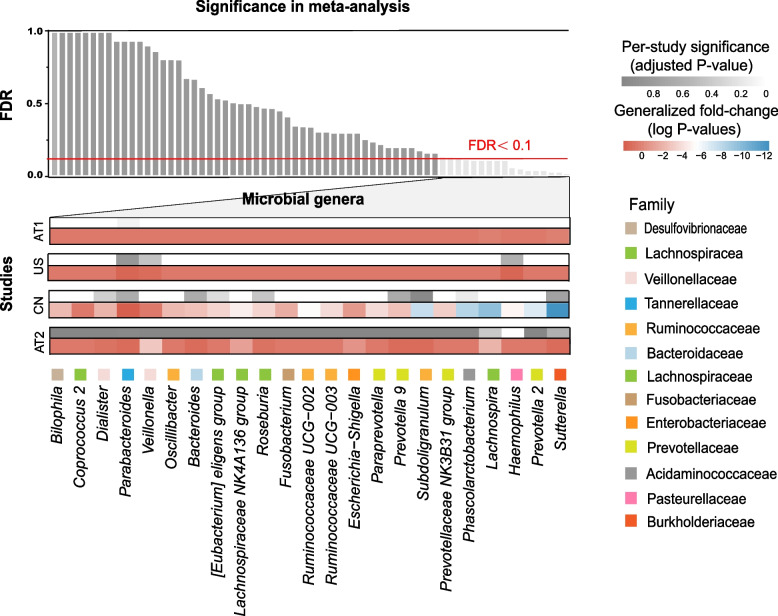
Twenty-three genera identified in relation to proton pump inhibitor (PPI) use in the meta-analysis

**Fig. 5:**
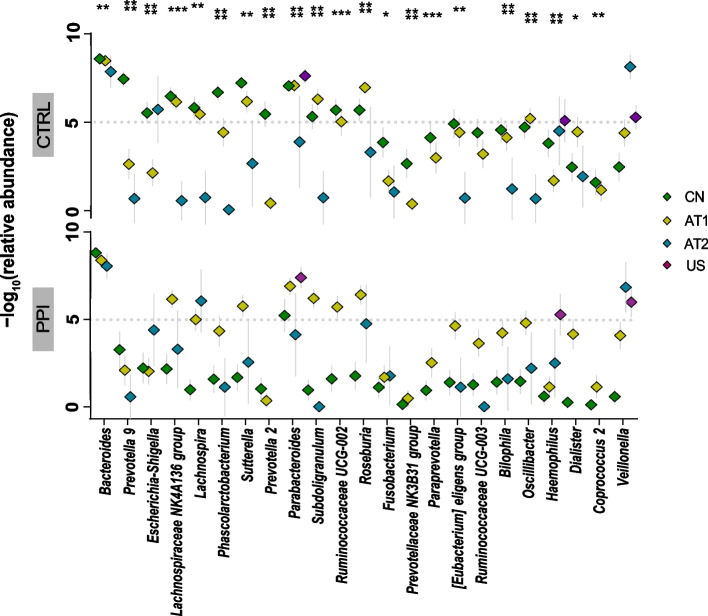
Distribution of the 23 differential microbial genera between the proton pump inhibitor (PPI) and control (CTRL) groups. The number of asterisks indicates the significance of the difference determined by the Wilcoxon test or Kruskal–Wallis test

We selected 77 genera with relative abundance greater than 0.1% from 315 genera detected in the PPI microbiome across studies. Among them, we surveyed a core group of the 23 most significant markers (FDR < 0.1) for further analysis, the significance of which is presented based on the adjusted *P*-value and generalized fold-change in Fig. 4. The results included multiple genera significantly associated with PPI use, such as *Parabacteroides*, *Veillonella*, *Bacteroides*, and *Prevotella 9*. Collectively, these 23 core microbial genera were mostly from the Lachnospiraceae, Prevotellaceae, Ruminococcaceae, and Veillonellaceae families. Compared with those in the participants of CTRL group, the abundances of the genera *Ruminococcaceae UCG-002*, *Subdoligranulum*, *Lachnospiraceae NK4A136 group*, *Roseburia*, *Paraprevotella*, and *Prevotella 2* were decreased in PPI users. These less-abundant genera mostly belonged to the Ruminococcaceae, Lachnospiraceae, and Prevotellaceae families. Moreover, the relative abundance of *Haemophilus* increased in PPI users. The family-level classification of the differential bacteria is provided in Fig. 4. The relative abundances of core microbial genera between the PPI and CTRL groups are shown in Fig. 5.

**Fig. 6:**
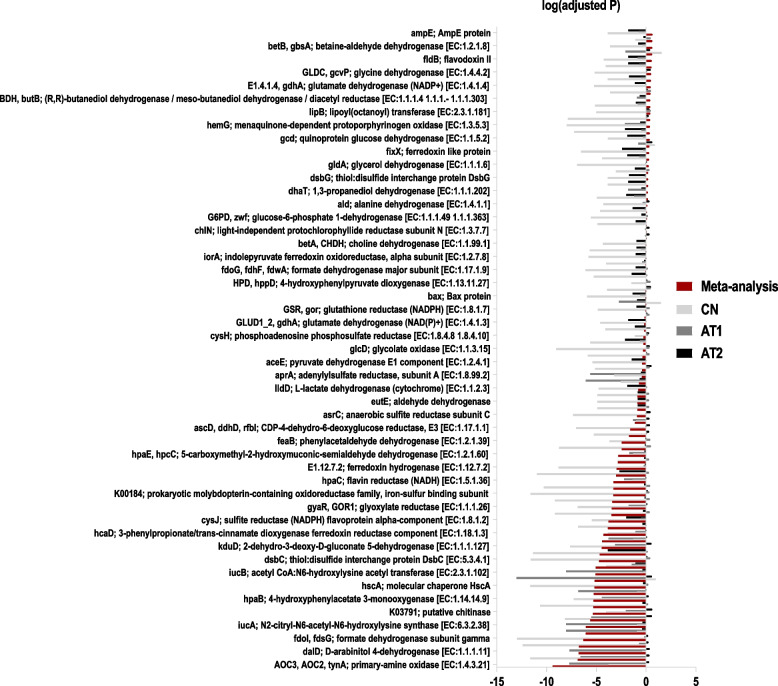
Overview of significantly different functional genes associated with PPI usage

PPI use was also closely associated with functional changes in the human gut microbiome. Referring to the KEGG database and based on 16S rRNA sequences, we applied PICRUSt2 to acquire functional gene information for the remaining three studies. Finally, 97 functional genes were identified with significant differences (adjusted *P* < 0.05, Wilcoxon tests) in the meta-analysis (Fig. 6, Table S3). These genes mainly encoded primary-amine oxidase, ferredoxin hydrogenase large subunit, D-arabinitol 4-dehydrogenase, and the fumarate reductase flavoprotein subunit, among others. Furthermore, we identified the top 20 enriched microbial functional pathways (Figure S1), which included glycolysis/gluconeogenesis, pyruvate metabolism, amino sugar and nucleotide sugar metabolism, and fructose and mannose metabolism. Our analysis also showed that compared with those in the CTRL group participants, the relative abundances of several functional genes related to the aforementioned metabolic pathways increased in PPI users. These included those encoding fbp (fructose-1,6-bisphosphatase I) [EC:3.1.3.11], frdA (fumarate reductase flavoprotein subunit) [EC:1.3.5.4], and frdC (fumarate reductase subunit C) (Figure S2).

The false discovery rate (FDR) of the identified 77 microbial genera in the meta-analysis is given by the bar height. The FDR-corrected *P*-values and generalized fold-change values of each differential genera within every study are presented as heatmaps in gray and as a color, respectively. Family-level taxonomic information is color-coded above the genus name and listed on the right side.

Bar heights show the significance of each functional gene expressed in each study. Bar colors indicate the distinction between single studies and meta-analyses.

### Gut microbiome markers related to PPI use

**Fig. 7:**
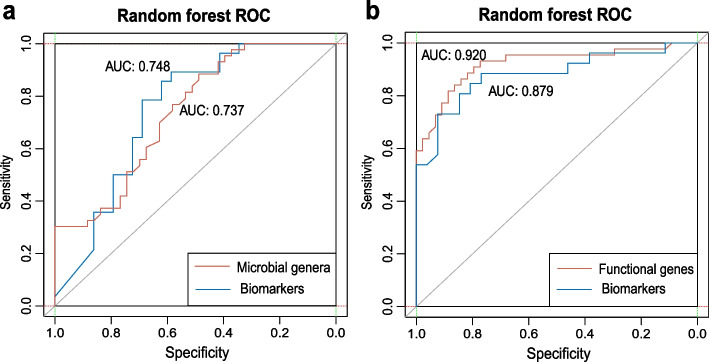
Classification of gut microbiota and functional genes associated with proton pump inhibitor (PPI) use based on the random forest model

To further excavate biomarkers in relation to the gut microbiota after PPI use, we generated a random forest classification model to evaluate gut microbiota taxonomic community composition and functional genes. Here, 77 microbial genera (relative abundance more than 0.1%) and 97 differential functional genes were selected as the data for model construction. Based on the top 30 discriminatory predictor variables (Figure S3), which were selected according to the importance score, six genera and twenty genes were finally chosen as gut microbiota composition biomarkers and functional biomarkers (adjusted *P* < 0.05, Wilcoxon tests, Figure S2). The six genera included *Phascolarctobacterium*, *Subdoligranulum*, *Sutterella*, *Lachnospiraceae UCG-010*, *Prevotella 2*, and *Prevotella 9*, whereas the 20 genes included those encoding AOC3, AOC2, tynA (primary-amine oxidase) [EC:1.4.3.21], fbp (fructose-1,6-bisphosphatase I) [EC:3.1.3.11], frdA (fumarate reductase flavoprotein subunit) [EC:1.3.5.4], and frdC (fumarate reductase subunit C), most of which are involved in citrate cycle, oxidative phosphorylation, pyruvate metabolism, and biosynthesis of secondary metabolites. Interestingly, we found that the abundance of these biomarkers was higher in the gut microbiota of the CTRL group participants. The performance of the model was quantified based on the AUC. The AUC was 0.748 for the six biomarker genera, compared with 0.737 for all microbial genera (Fig. 7a). Similarly, the AUC was 0.879 for the 20 biomarker genes and 0.920 of all genes (Fig. 7b). Thus, we verified the performance of the generated random forest model for classifying and identified biomarkers using this supervised learning technique, that showed better classification for functional genes. Overall, we proved the classification ability of random forest classifiers based on the gut microbiota as it relates to PPI use.

The receiver operating characteristic (ROC) curve in blue shows the area under the ROC (AUC) of biomarkers. The ROC curve in red shows the AUC of all microbial genera and genes.

## Discussion

Alterations in the intestinal microbiota with PPI usage could play a vital role in determining the potential associations between PPI use and liver cirrhosis, CDI, and other long-known adverse reactions [41–43]. In this study, we combined univariate analysis and a supervised classification method to identify core biomarkers of PPI-related gut microbiota. We have summarized and shown the influence of a commonly used drug type, namely PPIs, on the human gut microbiome composition and function.

We observed a lower alpha diversity after PPI use. In terms of the overall microbial structure, we found that the overall structure of the gut ecosystem was altered by the use of PPIs, which is in agreement with the results of several previous studies [25, 44–46]. Tsuda et al. (2015) and Kim et al. (2021) also reported significant differences in the gut microbiota between PPI users and non-users. It is speculated that this situation could be ascribed to the irreversible inactivation of pump molecules pumping out H ions, which results in the long-lasting inhibition of gastric acid secretion and disruption of the gastric acid barrier, thus altering the microbiota [47, 48]. Despite some variation, our results revealed some consistent changes in the gut microbiota after PPI use. We observed a decrease in genera from the Ruminococcaceae and Lachnospiraceae families in the meta-analysis, which has been previously reported in studies informing that PPI use could increase the risk of hepatic encephalopathy and liver cirrhosis [48–52]. These studies reported that the progression of liver diseases may accompany a decrease in *Ruminococcus*. This unfavorable situation could be the consequence of PPI-induced increase in hydrochloride salts, which will lead to a reduced pH environment that restrains the growth of butyric acid-producing bacteria, such as members of the Ruminococcaceae and Lachnospiraceae families [48]. In our meta-analysis, we also found the significant difference of *Haemophilus* between PPI and CTRL group, which is consistent with Wellhöner et al. and they found that the relative abundance of *Streptococcus spp.*, *Enterobacter spp.* and *Haemophilus spp.* was significantly increased in patients with PPI use irrespectively of the stage of liver disease [22]. Furthermore, it has been reported that a member of the Veillonellaceae family, *Veillonella*, tends to increase in abundance in conjunction with lactate synthesis [48]. This might explain the increase in the abundance of the genus *Veillonella* in our results and therefore is relevant to different types of infection, including intestinal infection [53]. As is known, *Prevotella spp.* play a key role in regulating human microbiome health and are mostly associated with oral infections. In fact, a newly published study found that the relative abundance of *Prevotella copri* and *Ruminococcus gnavus* is inversely correlated with the duration of PPI use in patients with CDI [54], which supports the discovery of the decrease in the abundance of *Paraprevotella*, *Prevotella 2*, and *Prevotella 9* belonging to the Prevotellaceae family in our results. The altered abundance of *Prevotella* caused by the use of PPIs might therefore worsen infection and be associated with some risk factors for inflammatory diseases. Although Imhann and Hojo described an increase in Enterobacteriaceae in the gut microbiota of PPI users in three cohorts, we were unable to replicate this phenomenon in the meta-analysis [2, 55]. This could be related to differences in the study subjects; specifically, the mentioned study [2] included healthy individuals and an IBS cohort.

After analyzing the diversity and composition of the gut microbiota, functional profiling was performed using PICRUSt. We found that PPI-associated gut microbiota functional biomarkers were highly enriched in carbohydrate metabolic pathways, such as glycolysis/gluconeogenesis, pyruvate metabolism, amino sugar and nucleotide sugar metabolism, and fructose and mannose metabolism. These findings were consistent with previous functional predictions to some extent [45, 56]. Shi et al. also reported that the pathways related to amino sugar and nucleotide sugar metabolism, sphingolipid metabolism, and fructose and mannose metabolism were more prevalent in PPI users. The important role played by pyruvate metabolism and gluconeogenesis is imperative to maintain the hepatic TCA cycle function and oxidation, biosynthesis, and antioxidant defense [57–59]. Our results thus illuminate the biological mechanisms underlying the effects of PPI use.

Supervised learning techniques have been employed as a classifier tool in many fields including food contamination detection, disease classification, and data classification [60–63]. The random forest model has been validated as an applicable model for excavating microbiome data [38, 64, 65]. Using the random forest model, Qian Li proved that changes in the gut microbiota could be used to identify individuals with a high risk of Type 2 diabetes, since the intestinal mucosal barrier is essential for improving insulin sensitivity and preventing the development of diabetes [66]. Pan et al. (2020) also analyzed the potential value of the intestinal microbiome as a biomarker in patients with schizophrenia, which could provide clues for targeted intervention for this disease. Here, we employed this method to identify biomarkers associated with PPI use. Based on AUC, we found that regarding functional genes as biomarkers related to PPI use is more accurate compared to microbial genera. The classification results by random forest model were consistent with the preliminary results of 23 differentially abundant bacteria and 97 differentially abundant functional genes screened before using the univariate and Wilcoxon tests. This provides robust support for the future use of random forest models to identify bacterial taxa and functional genes in relation to drug use.

This study has some limitations. First, age, bmi, gender and geography have big impact on the gut microbiome. Among the four included studies in our meta-analysis, one involved infant while the rest involved adults (Table S2). It takes time for the gut microbiome develops over the course of host infancy to eventually reach its adult form [67, 68]. Moreover, as none of the four studies provides age or gender information of each subject, potential confounding of individual microbial genera associations by patient demographics could not be calculated through mathematic method as Wirbel et al. have done [33]. Second, types of study design were not identical across the four included studies. Specifically, the design of two studies is self-control (Table 1), which means the comparison of influences of PPI on gut microbiota is based on patients with acidity issues rather than healthy individuals in the case of other two studies. Finally, the performance of the gut microbiota-based classification model based on PPI use needs to be validated in more datasets and with different populations worldwide.

Our meta-analysis revealed that gut microbiota dysbiosis induced by PPI use has a certain pattern and is closely associated with related complications. The random forest classification model provided strong support for the results of the identified differential genera and functional genes using univariate analysis. Altogether, our meta-analysis reflects significant effect of PPI use on gut microbiota homeostasis and helps to clarify the potential mechanisms underlying its side effects.

## Supplementary Information

**Additional file 1: Figure S1.** Metabolic pathways from KEGG database of differential functional genes.** Figure S2.** Six genera and 20 genes biomarkers in PPI group across CTRL group. The bars in red and grey show the relative abundance of the 6 genera and 20 genes in the CTRL and PPI group respectively.** Figure S3.** The importance score of the 30 discriminatory genera and genes.** Figure S4.** Classification of gut microbiota and functional genes associated with proton pump inhibitor (PPI) use based on the random forest model in every included study.** Table S1.** PubMed results of the meta-analysis search and reasons for exclusion of studies.** Table S2.** Baseline characteristics of study subjects.** Table S3.** Differential Metabolic Potential from PICRUSt2 of 16S rDNA based bacterial profile.

## Data Availability

Raw data for this meta-analysis are available online (see Table S1). The relative abundance table analysed during this study is included in the supplementary information files.
