# Supplementary material for: Meta-analysis of the effects of proton pump inhibitors on the human gut microbiota
Source: BMC Microbiol. 2023 Jun 19;23:171. doi: 10.1186/s12866-023-02895-w (PMC10278323; doi:10.1186/s12866-023-02895-w)
Supplement: Supplementary file 1 — Additional file 1: Figure S1. Metabolic pathways from KEGG database of differential functional genes. Figure S2. Six genera and 20 genes biomarkers in PPI group across CTRL group. The bars in red and grey show the relative abundance of the 6 genera and 20 genes in the CTRL and PPI group respectively. Figure S3. The importance score of the 30 discriminatory genera and genes. Figure S4. Classification of gut microbiota and functional genes associated with proton pump inhibitor (PPI) use based on the random forest model in every included study. Table S1. PubMed results of the meta-analysis search and reasons for exclusion of studies. Table S2. Baseline characteristics of study subjects. Table S3. Differential Metabolic Potential from PICRUSt2 of 16S rDNA based bacterial profile. [file 12866_2023_2895_MOESM1_ESM.docx]

**Appendix**


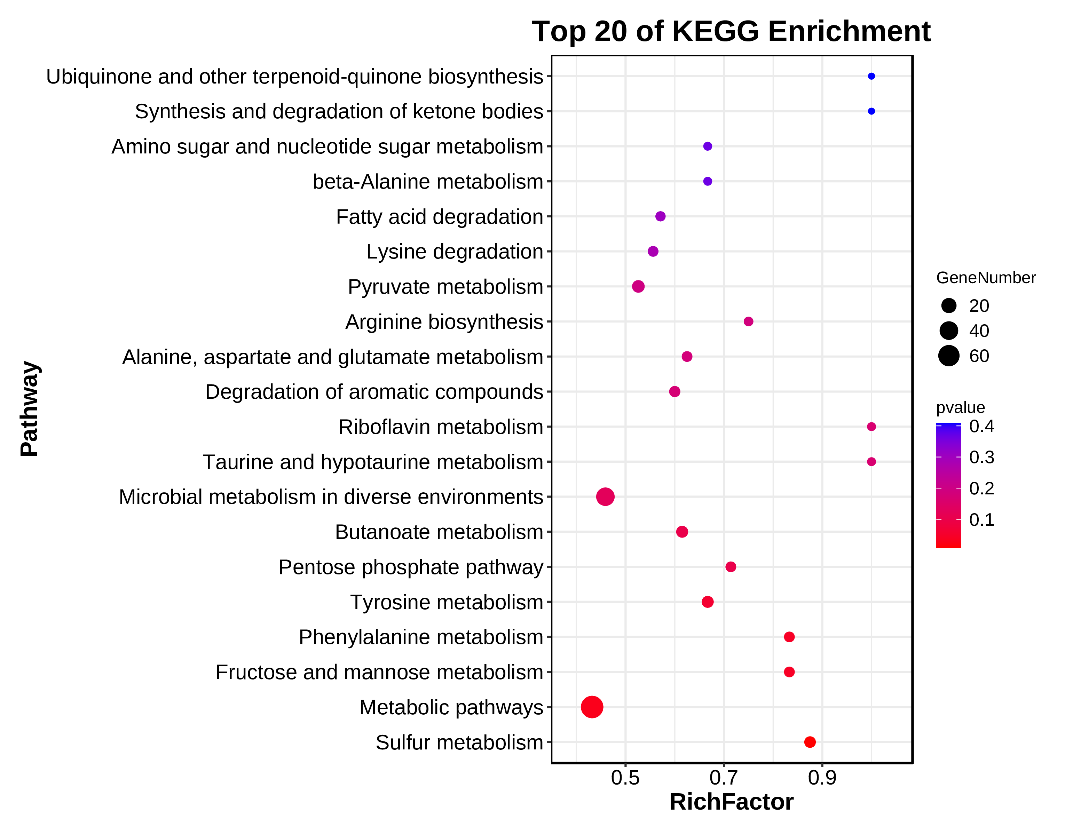


Figure S1 Metabolic pathways from KEGG database of differential functional genes.


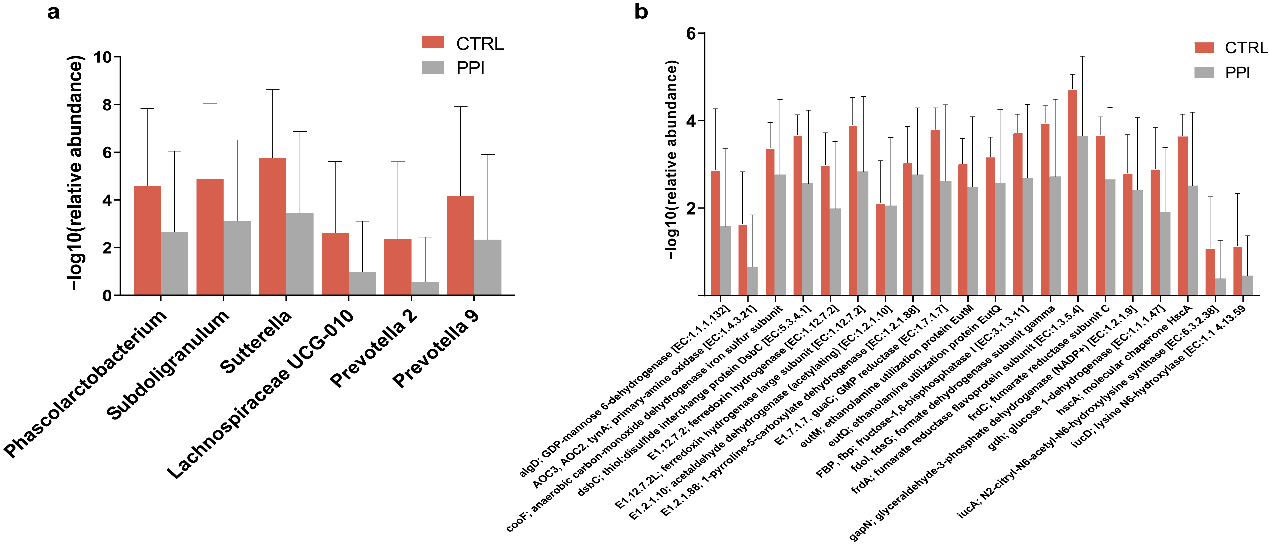


Figure S2. Six genera and 20 genes biomarkers in PPI group across CTRL group.

The bars in red and grey show the relative abundance of the 6 genera and 20 genes in the CTRL and PPI group respectively.


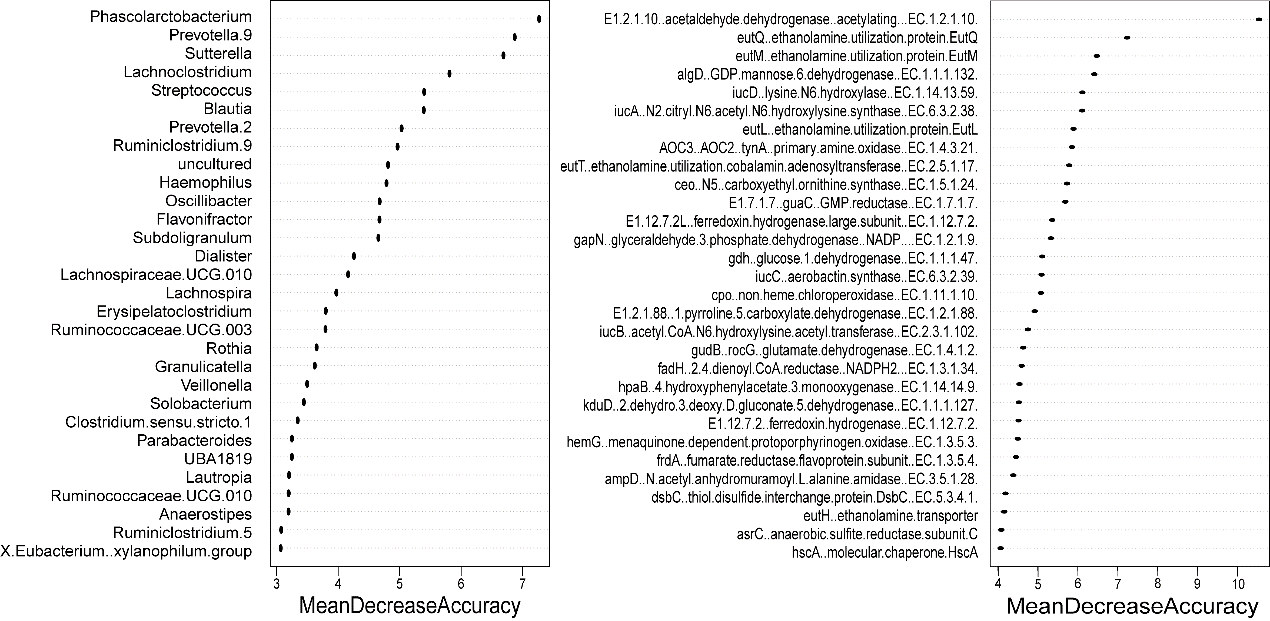


Figure S3 The importance score of the 30 discriminatory genera and genes.


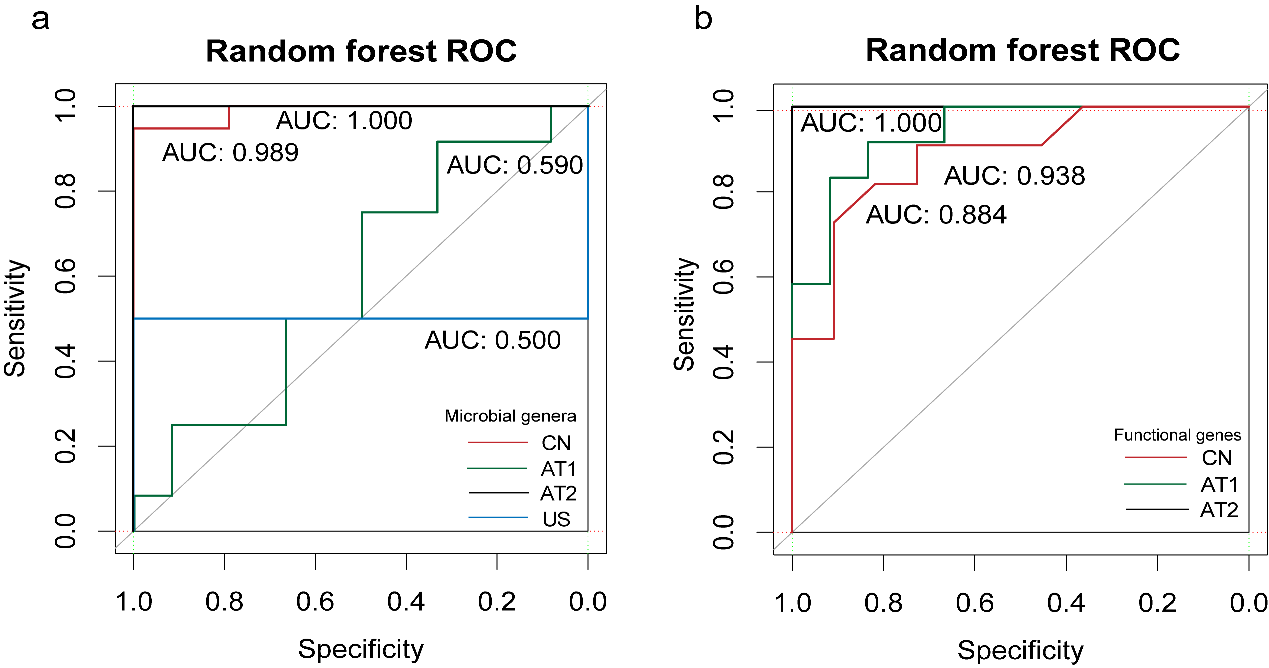


Figure S4 Classification of gut microbiota and functional genes associated with proton pump inhibitor (PPI) use based on the random forest model in every included study.

Table S1 PubMed results of the meta-analysis search and reasons for exclusion of studies.

| **Authors** | **Title** | **Year** | **doi** | **Exclusion Reason** |
| --- | --- | --- | --- | --- |
| MA Jackson et al. | Proton pump inhibitors alter the composition of the gut microbiota | 2016 | <http://orcid.org/0000-0002-7891-6217> | No raw reads |
| T Takagi et al. | The influence of long-term use of proton pump inhibitors on the gut microbiota: an age-sex-matched case-control study | 2018 | <https://doi.org/10.3164/jcbn.17-78> | Data not accessible |
| F Imhann et al. | The influence of proton pump inhibitors and other commonly used medication on the gut microbiota | 2017 | <https://doi.org/10.1080/19490976.2017.1284732> | No raw reads |
| LMM Gommers et al. | Low gut microbiota diversity and dietary magnesium intake are associated with the development of PPI-induced hypomagnesemia | 2019 | <https://doi.org/10.1096/fj.201900839R> | Data not accessible |
| EK Ward et al. | The effect of PPI use on human gut microbiota and weight loss in patients undergoing laparoscopic Roux-en-Y gastric bypass | 2014 | <https://doi.org/10.1007/s11695-014-1275-1> | Data not accessible |
| M Hojo et al | Gut Microbiota Composition Before and After Use of Proton Pump Inhibitors | 2018 | https://doi.org/10.1007/s10620-018-5122-4 | Data not accessible |
| [KR Reveles et al](https://scholar.google.com/citations?user=gOEoypkAAAAJ&hl=zh-CN&oi=sra). | Proton pump inhibitor use associated with changes in gut microbiota composition | 2018 | <http://dx.doi.org/10.1136/gutjnl-2017-315306> | Letter |
| JS Bajaj et al. | Proton Pump Inhibitor Initiation and Withdrawal affects Gut Microbiota and Readmission Risk in Cirrhosis | 2018 | 10.1038/s41395-018-0085-9 | No raw reads |
| Y Wang et al. | Advances in gut microbiota of viral hepatitis cirrhosis | 2019 | <https://doi.org/10.1155/2019/9726786> | Unrelated |
| JW Kim et al. | Influence of proton pump inhibitor or rebamipide use on gut microbiota of rheumatoid arthritis patients | 2021 | https: //doi.org/10.1093/rheumatology/keaa316 | No raw reads |
| D.G. Burke et al. | The altered gut microbiota in adults with cystic fibrosis | 2017 | <https://doi.org/10.1186/s12866-017-0968-8> | 454-pyrosequencing |
| AC Erber et al. | The role of gut microbiota, butyrate and proton pump inhibitors in amyotrophic lateral sclerosis: a systematic review | 2019 | <https://doi.org/10.1080/00207454.2019.1702549> | Review |
| L Simakachorn et al. | Gut Microbiota Characteristics in Children After the Use of Proton Pump Inhibitors | 2021 | 10.5152/tjg.2020.20245 | No raw reads |
| A G Clooney et al. | A comparison of the gut microbiome between long-term users and non-users of proton pump inhibitors | 2016 | [10.1111/apt.13568](https://doi.org/10.1111/apt.13568) | No raw reads |
| Singh et al. | Proton Pump Inhibitors: Risks and Rewards and Emerging Consequences to the Gut Microbiome | 2018 | https://doi.org/10.1002/ncp.10181 | Review |
| R K Weersma et al. | Interaction between drugs and the gut microbiome | 2020 | 10.1136/gutjnl-2019-320204 | No PPI samples |
| Shi et al. | Effects of Proton Pump Inhibitors on the Gastrointestinal Microbiota in Gastroesophageal Reflux Disease | 2019 | <https://doi.org/10.1016/j.gpb.2018.12.004> | The author did not respond for incomplete raw reads |
| Mishiro et al. | Oral microbiome alterations of healthy volunteers with proton pump inhibitor | 2017 | <https://doi.org/10.1111/jgh.14040> | No raw reads |
| Yuji Naito et al. | Intestinal Dysbiosis Secondary to Proton-Pump Inhibitor Use | 2018 | 10.1159/000481813 | Review |
| Lin et al. | Proton Pump Inhibitor-Induced Gut Dysbiosis Increases Mortality Rates for Patients with Clostridioides difficile Infection | 2022 | <https://doi.org/10.1128/spectrum.00486-22> | Not published when searching |
| A Tsuda et al. | Influence of Proton-Pump Inhibitors on the Luminal Microbiota in the Gastrointestinal Tract | 2015 | [10.1038/ctg.2015.20](https://doi.org/10.1038%2Fctg.2015.20) | Data not accessible |

Table S2 Baseline characteristics of study subjects.

| **Reference** | **Country** | **Age(years)** | | **Gender** | | **BMI** |
| --- | --- | --- | --- | --- | --- | --- |
|  |  | PPI | CTRL | male | female |  |
| Horvath et al. | Austria | 58(56-62) | 55(53-60) | 23 | 77 | NA |
| Castellani et al. | Austria | 0.43(0.042-0.85) | / | 8 | 4 | NA |
| Lin et al. | China | 68.3(53.5-76.5) | 64.1(53.1-75.1) | NA | NA | NA |
| Freedberg et al. | USA | 39.5(29.0-51.5) | / | 3 | 9 | 28.4(21.3-34.5) |

NA means relevant information was not reported in the included studies. Data is given as median and range.

Table S3 Differential Metabolic Potential from PICRUSt2 of 16S rDNA based bacterial profile.

|  |  | p value | | | | p. adjusted | | | | log2FC | | | |
| --- | --- | --- | --- | --- | --- | --- | --- | --- | --- | --- | --- | --- | --- |
| K_ID | Description | Meta | CN | AT1 | AT2 | Meta | CN | AT1 | AT2 | Meta | CN | AT1 | AT2 |
| K00276 | AOC3, AOC2, tynA; primary-amine oxidase [EC:1.4.3.21] | 4.42E-12 | 3.01E-06 | 5.91E-09 | 0.4701 | 4.29E-10 | 4.23E-06 | 9.93E-08 | 0.599156 | -9.38741 | -3.75832 | -7.71678 | 0.311795 |
| K00533 | E1.12.7.2L; ferredoxin hydrogenase large subunit [EC:1.12.7.2] | 1.43E-09 | 3.97E-14 | 7.88E-08 | 0.513723 | 4.67E-08 | 7.2E-13 | 9.55E-07 | 0.599156 | -6.87751 | -11.638 | -6.59174 | 0.350333 |
| K00007 | dalD; D-arabinitol 4-dehydrogenase [EC:1.1.1.11] | 1.77E-09 | 0.007151 | 6.14E-09 | 0.540279 | 4.67E-08 | 0.007973 | 9.93E-08 | 0.616554 | -6.78446 | -0.38208 | -7.69992 | 0.372223 |
| K00244 | frdA; fumarate reductase flavoprotein subunit [EC:1.3.5.4] | 1.93E-09 | 6.85E-15 | 0.061625 | 0.40951 | 4.67E-08 | 3.32E-13 | 0.184765 | 0.544144 | -6.7484 | -12.4007 | -0.69833 | 0.251869 |
| K00127 | fdoI, fdsG; formate dehydrogenase subunit gamma | 5.36E-09 | 1.84E-15 | 0.203551 | 0.377685 | 1.04E-07 | 1.78E-13 | 0.411342 | 0.508826 | -6.3034 | -12.9725 | -0.17942 | 0.216735 |
| K03895 | iucC; aerobactin synthase [EC:6.3.2.39] | 9.31E-09 | 0.00219 | 2.84E-09 | 0.088734 | 1.3E-07 | 0.00247 | 6.88E-08 | 0.195618 | -6.06381 | -0.89606 | -8.03547 | -0.41231 |
| K03894 | iucA; N2-citryl-N6-acetyl-N6-hydroxylysine synthase [EC:6.3.2.38] | 9.41E-09 | 0.00219 | 2.84E-09 | 0.10053 | 1.3E-07 | 0.00247 | 6.88E-08 | 0.203155 | -6.05906 | -0.89606 | -8.03547 | -0.3581 |
| K00034 | gdh; glucose 1-dehydrogenase [EC:1.1.1.47] | 2.61E-08 | 1.19E-10 | 9.54E-07 | 0.932301 | 3.16E-07 | 7.18E-10 | 8.42E-06 | 0.951928 | -5.61701 | -8.16268 | -5.50841 | 0.60916 |
| K03791 | K03791; putative chitinase | 4.88E-08 | 1.63E-06 | 0.00288 | 1 | 4.63E-07 | 2.6E-06 | 0.014705 | 1 | -5.34456 | -4.0232 | -2.02864 | 0.639604 |
| K03806 | ampD; N-acetyl-anhydromuramoyl-L-alanine amidase [EC:3.5.1.28] | 5.27E-08 | 3.88E-13 | 0.506599 | 0.10053 | 4.63E-07 | 4.18E-12 | 0.692114 | 0.203155 | -5.31115 | -10.6477 | 0.216575 | -0.3581 |
| K00483 | hpaB; 4-hydroxyphenylacetate 3-monooxygenase [EC:1.14.14.9] | 5.66E-08 | 8.92E-10 | 1.1E-05 | 0.452822 | 4.63E-07 | 4.12E-09 | 8.86E-05 | 0.58565 | -5.27987 | -7.28602 | -4.44805 | 0.295532 |
| K03897 | iucD; lysine N6-hydroxylase [EC:1.14.13.59] | 5.73E-08 | 0.00219 | 4.88E-08 | 0.26567 | 4.63E-07 | 0.00247 | 6.77E-07 | 0.390454 | -5.27473 | -0.89606 | -6.79942 | 0.063946 |
| K04044 | hscA; molecular chaperone HscA | 7.05E-08 | 4.09E-14 | 0.545253 | 0.113502 | 5E-07 | 7.2E-13 | 0.724514 | 0.220195 | -5.1848 | -11.6246 | 0.248509 | -0.30539 |
| K00066 | algD; GDP-mannose 6-dehydrogenase [EC:1.1.1.132] | 7.22E-08 | 0.174104 | 2.97E-14 | 0.904603 | 5E-07 | 0.177769 | 2.88E-12 | 0.933473 | -5.17416 | 1.00438 | -13.0158 | 0.596062 |
| K03896 | iucB; acetyl CoA:N6-hydroxylysine acetyl transferase [EC:2.3.1.102] | 8.91E-08 | 0.008924 | 2.84E-09 | 0.10053 | 5.65E-07 | 0.009836 | 6.88E-08 | 0.203155 | -5.08314 | -0.28589 | -8.03547 | -0.3581 |
| K03893 | arsB; arsenical pump membrane protein | 9.32E-08 | 1.6E-06 | 0.011025 | 0.019347 | 5.65E-07 | 2.59E-06 | 0.046495 | 0.069885 | -5.06337 | -4.03179 | -1.44573 | -1.07378 |
| K03981 | dsbC; thiol:disulfide interchange protein DsbC [EC:5.3.4.1] | 2.01E-07 | 4.45E-14 | 0.880287 | 0.218921 | 1.15E-06 | 7.2E-13 | 0.948754 | 0.331802 | -4.72997 | -11.5876 | 0.456535 | -0.02011 |
| K00364 | E1.7.1.7, guaC; GMP reductase [EC:1.7.1.7] | 2.37E-07 | 7.75E-14 | 0.460386 | 3.33E-05 | 1.27E-06 | 1.07E-12 | 0.655676 | 0.003228 | -4.65889 | -11.3472 | 0.175033 | -3.83818 |
| K00065 | kduD; 2-dehydro-3-deoxy-D-gluconate 5-dehydrogenase [EC:1.1.1.127] | 3.63E-07 | 4.04E-10 | 0.000241 | 0.842836 | 1.85E-06 | 2.06E-09 | 0.001672 | 0.879087 | -4.47294 | -7.62979 | -3.1055 | 0.565348 |
| K00455 | hpaD, hpcB; 3,4-dihydroxyphenylacetate 2,3-dioxygenase [EC:1.13.11.15] | 4.09E-07 | 0.000833 | 5.27E-05 | 0.214095 | 1.98E-06 | 0.00101 | 0.000393 | 0.331802 | -4.42154 | -1.31561 | -3.76664 | -0.02979 |
| K00529 | hcaD; 3-phenylpropionate/trans-cinnamate dioxygenase ferredoxin reductase component [EC:1.18.1.3] | 5.5E-07 | 2.55E-06 | 0.026916 | 0.193834 | 2.54E-06 | 3.64E-06 | 0.100418 | 0.308228 | -4.29221 | -3.83007 | -1.05808 | -0.07297 |
| K00219 | fadH; 2,4-dienoyl-CoA reductase (NADPH2) [EC:1.3.1.34] | 1.59E-06 | 2.47E-09 | 0.248914 | 0.10053 | 6.99E-06 | 9.21E-09 | 0.446183 | 0.203155 | -3.83244 | -6.84395 | -0.09204 | -0.3581 |
| K00380 | cysJ; sulfite reductase (NADPH) flavoprotein alpha-component [EC:1.8.1.2] | 1.77E-06 | 6.05E-08 | 0.096482 | 0.002316 | 7.47E-06 | 1.68E-07 | 0.239968 | 0.023517 | -3.78456 | -5.45468 | -0.50364 | -1.99558 |
| K00246 | frdC; fumarate reductase subunit C | 3.61E-06 | 1.18E-11 | 0.753859 | 0.113502 | 1.43E-05 | 9.52E-11 | 0.84051 | 0.220195 | -3.47555 | -9.16549 | 0.389201 | -0.30539 |
| K00015 | gyaR, GOR1; glyoxylate reductase [EC:1.1.1.26] | 3.68E-06 | 2.3E-09 | 0.005394 | 0.218921 | 1.43E-05 | 8.92E-09 | 0.024917 | 0.331802 | -3.46732 | -6.87489 | -1.75615 | -0.02011 |
| K00245 | frdB; fumarate reductase iron-sulfur subunit [EC:1.3.5.4] | 4.39E-06 | 1.18E-11 | 0.753859 | 0.159973 | 1.64E-05 | 9.52E-11 | 0.84051 | 0.277096 | -3.39049 | -9.16549 | 0.389201 | -0.15635 |
| K00184 | K00184; prokaryotic molybdopterin-containing oxidoreductase family, iron-sulfur binding subunit | 5.55E-06 | 4.23E-14 | 0.700123 | 0.37074 | 1.96E-05 | 7.2E-13 | 0.837536 | 0.506504 | -3.28855 | -11.6098 | 0.357085 | 0.208674 |
| K03841 | FBP, fbp; fructose-1,6-bisphosphatase I [EC:3.1.3.11] | 5.82E-06 | 8.98E-13 | 0.818394 | 0.143168 | 1.96E-05 | 8.71E-12 | 0.902094 | 0.262024 | -3.26762 | -10.2831 | 0.424873 | -0.20455 |
| K00484 | hpaC; flavin reductase (NADH) [EC:1.5.1.36] | 5.86E-06 | 0.000746 | 0.001889 | 0.193543 | 1.96E-05 | 0.000928 | 0.011454 | 0.308228 | -3.26513 | -1.36363 | -2.21179 | -0.07362 |
| K00260 | gudB, rocG; glutamate dehydrogenase [EC:1.4.1.2] | 1.02E-05 | 1.87E-13 | 0.619714 | 0.000496 | 3.3E-05 | 2.27E-12 | 0.791473 | 0.023517 | -3.02355 | -10.9635 | 0.304102 | -2.66532 |
| K00532 | E1.12.7.2; ferredoxin hydrogenase [EC:1.12.7.2] | 1.21E-05 | 2.74E-11 | 0.172153 | 0.178182 | 3.79E-05 | 1.9E-10 | 0.355294 | 0.297994 | -2.95 | -8.79879 | -0.25218 | -0.10953 |
| K00064 | E1.1.1.122; D-threo-aldose 1-dehydrogenase [EC:1.1.1.122] | 1.56E-05 | 2.54E-05 | 0.636442 | 0.150381 | 4.73E-05 | 3.28E-05 | 0.791473 | 0.270128 | -2.83945 | -2.83239 | 0.315669 | -0.1832 |
| K00151 | hpaE, hpcC; 5-carboxymethyl-2-hydroxymuconic-semialdehyde dehydrogenase [EC:1.2.1.60] | 1.68E-05 | 0.000833 | 0.006139 | 0.157123 | 4.94E-05 | 0.00101 | 0.027066 | 0.277096 | -2.80756 | -1.31561 | -1.70002 | -0.16416 |
| K00124 | fdoH, fdsB; formate dehydrogenase iron-sulfur subunit | 3.81E-05 | 3.1E-11 | 0.990151 | 0.178182 | 0.000107 | 2E-10 | 0.99803 | 0.297994 | -2.45154 | -8.74529 | 0.507612 | -0.10953 |
| K00146 | feaB; phenylacetaldehyde dehydrogenase [EC:1.2.1.39] | 3.86E-05 | 3.64E-06 | 0.833642 | 0.18846 | 0.000107 | 5.05E-06 | 0.908576 | 0.308228 | -2.44654 | -3.67522 | 0.43289 | -0.08518 |
| K00412 | CYTB, petB; ubiquinol-cytochrome c reductase cytochrome b subunit | 0.00021 | 9.8E-08 | 0.404833 | 0.485063 | 0.000567 | 2.5E-07 | 0.604029 | 0.599156 | -1.71011 | -5.24532 | 0.119187 | 0.325402 |
| K00523 | ascD, ddhD, rfbI; CDP-4-dehydro-6-deoxyglucose reductase, E3 [EC:1.17.1.1] | 0.00028 | 1.64E-09 | 0.260949 | 0.572605 | 0.000733 | 6.91E-09 | 0.452001 | 0.638422 | -1.58624 | -7.02201 | -0.07153 | 0.39746 |
| K00254 | DHODH, pyrD; dihydroorotate dehydrogenase [EC:1.3.5.2] | 0.000796 | 0.001721 | 0.015226 | 0.513723 | 0.002033 | 0.002012 | 0.061538 | 0.599156 | -1.13179 | -1.00055 | -1.30551 | 0.350333 |
| K00385 | asrC; anaerobic sulfite reductase subunit C | 0.001341 | 8.06E-10 | 0.357096 | 0.67066 | 0.003305 | 3.91E-09 | 0.541224 | 0.730944 | -0.90555 | -7.33015 | 0.064696 | 0.466107 |
| K04020 | eutD; phosphotransacetylase | 0.001431 | 2.11E-07 | 0.635405 | 0.033241 | 0.003305 | 4.35E-07 | 0.791473 | 0.089567 | -0.87718 | -4.91259 | 0.314962 | -0.83872 |
| K04021 | eutE; aldehyde dehydrogenase | 0.001431 | 2.11E-07 | 0.635405 | 0.033241 | 0.003305 | 4.35E-07 | 0.791473 | 0.089567 | -0.87718 | -4.91259 | 0.314962 | -0.83872 |
| K04025 | eutK; ethanolamine utilization protein EutK | 0.001431 | 2.11E-07 | 0.635405 | 0.033241 | 0.003305 | 4.35E-07 | 0.791473 | 0.089567 | -0.87718 | -4.91259 | 0.314962 | -0.83872 |
| K00101 | lldD; L-lactate dehydrogenase (cytochrome) [EC:1.1.2.3] | 0.001732 | 2.91E-07 | 0.287157 | 0.002914 | 0.003908 | 5.65E-07 | 0.48867 | 0.023517 | -0.79422 | -4.77204 | -0.02997 | -1.8959 |
| K00395 | aprB; adenylylsulfate reductase, subunit B [EC:1.8.99.2] | 0.002074 | 5.05E-05 | 2.64E-07 | 0.052891 | 0.004573 | 6.45E-05 | 2.84E-06 | 0.13155 | -0.71597 | -2.53294 | -6.06704 | -0.63701 |
| K00394 | aprA; adenylylsulfate reductase, subunit A [EC:1.8.99.2] | 0.004025 | 1.05E-05 | 7.6E-07 | 0.068723 | 0.008677 | 1.39E-05 | 7.38E-06 | 0.162588 | -0.42807 | -3.21535 | -5.60706 | -0.52329 |
| K00534 | E1.12.7.2S; ferredoxin hydrogenase small subunit [EC:1.12.7.2] | 0.004263 | 1.27E-07 | 0.751493 | 0.829031 | 0.008983 | 2.99E-07 | 0.84051 | 0.874087 | -0.40317 | -5.13189 | 0.387836 | 0.558175 |
| K00163 | aceE; pyruvate dehydrogenase E1 component [EC:1.2.4.1] | 0.004352 | 7.13E-08 | 0.251906 | 0.008293 | 0.008983 | 1.92E-07 | 0.446183 | 0.042339 | -0.39414 | -5.38321 | -0.08685 | -1.44168 |
| K00210 | E1.3.1.12; prephenate dehydrogenase [EC:1.3.1.12] | 0.004746 | 2.34E-08 | 0.716655 | 0.291343 | 0.00959 | 7.56E-08 | 0.837536 | 0.415592 | -0.35658 | -5.86772 | 0.367221 | 0.104009 |
| K00104 | glcD; glycolate oxidase [EC:1.1.3.15] | 0.0053 | 1.63E-11 | 0.709291 | 0.551167 | 0.010491 | 1.22E-10 | 0.837536 | 0.621665 | -0.30861 | -9.02437 | 0.362735 | 0.380888 |
| K03788 | aphA; acid phosphatase (class B) [EC:3.1.3.2] | 0.005687 | 4.22E-08 | 0.074852 | 0.00183 | 0.010926 | 1.24E-07 | 0.207447 | 0.023517 | -0.27795 | -5.6113 | -0.61388 | -2.098 |
| K00390 | cysH; phosphoadenosine phosphosulfate reductase [EC:1.8.4.8 1.8.4.10] | 0.005745 | 0.001313 | 0.532207 | 0.477575 | 0.010926 | 0.001573 | 0.717002 | 0.599156 | -0.2736 | -1.11809 | 0.237992 | 0.318646 |
| K00121 | frmA, ADH5, adhC; S-(hydroxymethyl)glutathione dehydrogenase / alcohol dehydrogenase [EC:1.1.1.284 1.1.1.1] | 0.007046 | 1.41E-06 | 0.903696 | 0.017271 | 0.012967 | 2.41E-06 | 0.951276 | 0.069804 | -0.18491 | -4.08599 | 0.467933 | -1.12307 |
| K00261 | GLUD1_2, gdhA; glutamate dehydrogenase (NAD(P)+) [EC:1.4.1.3] | 0.007085 | 4.32E-07 | 0.354524 | 0.003637 | 0.012967 | 8.07E-07 | 0.541224 | 0.023517 | -0.18254 | -4.60055 | 0.061556 | -1.7997 |
| K00045 | E1.1.1.67, mtlK; mannitol 2-dehydrogenase [EC:1.1.1.67] | 0.007428 | 0.015088 | 0.687361 | 0.310208 | 0.013342 | 0.016444 | 0.837536 | 0.43609 | -0.16202 | -0.0578 | 0.349096 | 0.131258 |
| K00383 | GSR, gor; glutathione reductase (NADPH) [EC:1.8.1.7] | 0.008789 | 2.44E-07 | 0.731462 | 0.024184 | 0.015297 | 4.82E-07 | 0.84051 | 0.073309 | -0.08891 | -4.84982 | 0.376102 | -0.97686 |
| K03933 | cpbD; chitin-binding protein | 0.008846 | 0.563684 | 0.000575 | 0.028421 | 0.015297 | 0.569556 | 0.003719 | 0.08354 | -0.08612 | 1.514608 | -2.7283 | -0.90676 |
| K03796 | bax; Bax protein | 0.008989 | 2.01E-08 | 0.0813 | 0.010045 | 0.015297 | 6.71E-08 | 0.219058 | 0.046396 | -0.07915 | -5.93411 | -0.578 | -1.35847 |
| K00019 | E1.1.1.30, bdh; 3-hydroxybutyrate dehydrogenase [EC:1.1.1.30] | 0.009161 | 2.07E-06 | 0.946517 | 0.728978 | 0.01532 | 3.25E-06 | 0.966785 | 0.777042 | -0.07094 | -3.91951 | 0.488039 | 0.502319 |
| K00457 | HPD, hppD; 4-hydroxyphenylpyruvate dioxygenase [EC:1.13.11.27] | 0.010609 | 0.00057 | 0.898654 | 0.224097 | 0.017209 | 0.000718 | 0.951276 | 0.334421 | -0.00719 | -1.48032 | 0.465503 | -0.00996 |
| K00381 | cysI; sulfite reductase (NADPH) hemoprotein beta-component [EC:1.8.1.2] | 0.010644 | 9.42E-08 | 0.300869 | 0.008293 | 0.017209 | 2.47E-07 | 0.503177 | 0.042339 | -0.00574 | -5.26217 | -0.00971 | -1.44168 |
| K00123 | fdoG, fdhF, fdwA; formate dehydrogenase major subunit [EC:1.17.1.9] | 0.011937 | 1.42E-08 | 0.466408 | 0.020489 | 0.018745 | 4.91E-08 | 0.655676 | 0.069885 | 0.044014 | -6.085 | 0.180677 | -1.04887 |
| K04047 | dps; starvation-inducible DNA-binding protein | 0.012135 | 1.59E-06 | 0.125225 | 0.143168 | 0.018745 | 2.59E-06 | 0.28921 | 0.262024 | 0.051166 | -4.03589 | -0.3904 | -0.20455 |
| K00179 | iorA; indolepyruvate ferredoxin oxidoreductase, alpha subunit [EC:1.2.7.8] | 0.012411 | 2.73E-08 | 0.104784 | 0.020489 | 0.018745 | 8.55E-08 | 0.254102 | 0.069885 | 0.06095 | -5.79976 | -0.46779 | -1.04887 |
| K00180 | iorB; indolepyruvate ferredoxin oxidoreductase, beta subunit [EC:1.2.7.8] | 0.012411 | 3.73E-08 | 0.092696 | 0.024184 | 0.018745 | 1.13E-07 | 0.239561 | 0.073309 | 0.06095 | -5.66479 | -0.52103 | -0.97686 |
| K00108 | betA, CHDH; choline dehydrogenase [EC:1.1.99.1] | 0.012689 | 7.71E-07 | 0.226246 | 0.024184 | 0.018745 | 1.38E-06 | 0.429453 | 0.073309 | 0.070557 | -4.3494 | -0.13351 | -0.97686 |
| K04040 | chlG, bchG; chlorophyll/bacteriochlorophyll a synthase [EC:2.5.1.62 2.5.1.133] | 0.012947 | 0.015576 | 0.314626 | 0.518856 | 0.018745 | 0.016603 | 0.508645 | 0.599156 | 0.07932 | -0.04397 | 0.009705 | 0.354652 |
| K04038 | chlN; light-independent protochlorophyllide reductase subunit N [EC:1.3.7.7] | 0.012947 | 0.015576 | 0.314626 | 0.518856 | 0.018745 | 0.016603 | 0.508645 | 0.599156 | 0.07932 | -0.04397 | 0.009705 | 0.354652 |
| K00285 | dadA; D-amino-acid dehydrogenase [EC:1.4.5.1] | 0.013214 | 2.19E-07 | 0.354402 | 0.020489 | 0.01885 | 4.43E-07 | 0.541224 | 0.069885 | 0.088179 | -4.89541 | 0.061407 | -1.04887 |
| K00036 | G6PD, zwf; glucose-6-phosphate 1-dehydrogenase [EC:1.1.1.49 1.1.1.363] | 0.013941 | 4.77E-08 | 0.500299 | 0.078024 | 0.01936 | 1.36E-07 | 0.692114 | 0.176008 | 0.111421 | -5.55771 | 0.211141 | -0.46817 |
| K00228 | CPOX, hemF; coproporphyrinogen III oxidase [EC:1.3.3.3] | 0.013971 | 4.18E-07 | 0.223275 | 0.010045 | 0.01936 | 7.95E-07 | 0.429453 | 0.046396 | 0.112358 | -4.615 | -0.13925 | -1.35847 |
| K00259 | ald; alanine dehydrogenase [EC:1.4.1.1] | 0.014897 | 8.66E-07 | 0.136549 | 0.513723 | 0.020352 | 1.53E-06 | 0.308029 | 0.599156 | 0.14023 | -4.29898 | -0.3528 | 0.350333 |
| K00247 | frdD; fumarate reductase subunit D | 0.017095 | 1.97E-07 | 0.019154 | 0.002316 | 0.02303 | 4.34E-07 | 0.074318 | 0.023517 | 0.199993 | -4.94259 | -1.20582 | -1.99558 |
| K00086 | dhaT; 1,3-propanediol dehydrogenase [EC:1.1.1.202] | 0.017435 | 0.017257 | 0.0047 | 0.078024 | 0.023116 | 0.018195 | 0.022794 | 0.176008 | 0.208566 | 0.000539 | -1.81602 | -0.46817 |
| K00427 | lldP, lctP; L-lactate permease | 0.017635 | 2.41E-06 | 0.230222 | 0.003637 | 0.023116 | 3.54E-06 | 0.429453 | 0.023517 | 0.213513 | -3.85409 | -0.12594 | -1.7997 |
| K03805 | dsbG; thiol:disulfide interchange protein DsbG | 0.018429 | 2.41E-06 | 0.218589 | 0.004513 | 0.023647 | 3.54E-06 | 0.429453 | 0.025751 | 0.232647 | -3.85409 | -0.14846 | -1.70593 |
| K00135 | gabD; succinate-semialdehyde dehydrogenase / glutarate-semialdehyde dehydrogenase [EC:1.2.1.16 1.2.1.79 1.2.1.20] | 0.018527 | 1.57E-05 | 0.694644 | 0.017271 | 0.023647 | 2.06E-05 | 0.837536 | 0.069804 | 0.234947 | -3.03958 | 0.353673 | -1.12307 |
| K00005 | gldA; glycerol dehydrogenase [EC:1.1.1.6] | 0.020679 | 2.03E-09 | 0.334368 | 0.291343 | 0.02605 | 8.2E-09 | 0.5317 | 0.415592 | 0.282663 | -6.92915 | 0.036136 | 0.104009 |
| K00138 | aldB; aldehyde dehydrogenase [EC:1.2.1.-] | 0.022844 | 6.42E-07 | 0.066707 | 0.002914 | 0.028274 | 1.18E-06 | 0.19031 | 0.023517 | 0.325902 | -4.42878 | -0.66392 | -1.8959 |
| K03855 | fixX; ferredoxin like protein | 0.023027 | 5.17E-09 | 0.157624 | 0.000858 | 0.028274 | 1.86E-08 | 0.332381 | 0.023517 | 0.329374 | -6.5227 | -0.29047 | -2.42694 |
| K00249 | ACADM, acd; acyl-CoA dehydrogenase [EC:1.3.8.7] | 0.02375 | 0.140547 | 0.050626 | 1 | 0.028797 | 0.145033 | 0.163692 | 1 | 0.342804 | 0.911395 | -0.78371 | 0.639604 |
| K00117 | gcd; quinoprotein glucose dehydrogenase [EC:1.1.5.2] | 0.026196 | 2.55E-06 | 0.155135 | 0.002914 | 0.03137 | 3.64E-06 | 0.332381 | 0.023517 | 0.385363 | -3.83007 | -0.29738 | -1.8959 |
| K00313 | fixC; electron transfer flavoprotein-quinone oxidoreductase [EC:1.5.5.-] | 0.026781 | 1.03E-09 | 0.107994 | 0.00183 | 0.031496 | 4.55E-09 | 0.255498 | 0.023517 | 0.394966 | -7.2232 | -0.45469 | -2.098 |
| K00230 | hemG; menaquinone-dependent protoporphyrinogen oxidase [EC:1.3.5.3] | 0.02695 | 1.81E-10 | 0.002295 | 0.059657 | 0.031496 | 1.03E-09 | 0.013095 | 0.144668 | 0.397701 | -7.97817 | -2.1273 | -0.58473 |
| K00248 | ACADS, bcd; butyryl-CoA dehydrogenase [EC:1.3.8.1] | 0.028327 | 2.44E-10 | 0.430937 | 0.442833 | 0.032711 | 1.31E-09 | 0.623894 | 0.580471 | 0.419338 | -7.84967 | 0.146324 | 0.285845 |
| K03801 | lipB; lipoyl(octanoyl) transferase [EC:2.3.1.181] | 0.02927 | 1.69E-07 | 0.946851 | 0.513723 | 0.033275 | 3.81E-07 | 0.966785 | 0.599156 | 0.433556 | -5.00873 | 0.488193 | 0.350333 |
| K00098 | idnD; L-idonate 5-dehydrogenase [EC:1.1.1.264] | 0.029502 | 1.29E-07 | 0.044532 | 0.020894 | 0.033275 | 2.99E-07 | 0.150972 | 0.069885 | 0.43698 | -5.12482 | -0.83942 | -1.04038 |
| K00004 | BDH, butB; (R,R)-butanediol dehydrogenase / meso-butanediol dehydrogenase / diacetyl reductase [EC:1.1.1.4 1.1.1.- 1.1.1.303] | 0.030112 | 0.01961 | 0.03651 | 0.051865 | 0.033574 | 0.020454 | 0.131166 | 0.13155 | 0.44588 | 0.056059 | -0.92567 | -0.64552 |
| K03820 | lnt; apolipoprotein N-acyltransferase [EC:2.3.1.-] | 0.031136 | 4.19E-06 | 0.99803 | 0.347358 | 0.03432 | 5.64E-06 | 0.99803 | 0.481339 | 0.460393 | -3.61426 | 0.511054 | 0.180382 |
| K00262 | E1.4.1.4, gdhA; glutamate dehydrogenase (NADP+) [EC:1.4.1.4] | 0.031844 | 1.02E-07 | 0.252991 | 0.017271 | 0.034707 | 2.53E-07 | 0.446183 | 0.069804 | 0.470169 | -5.22827 | -0.08499 | -1.12307 |
| K00216 | entA; 2,3-dihydro-2,3-dihydroxybenzoate dehydrogenase [EC:1.3.1.28] | 0.033845 | 2.41E-06 | 0.093849 | 0.004513 | 0.036478 | 3.54E-06 | 0.239561 | 0.025751 | 0.496631 | -3.85409 | -0.51566 | -1.70593 |
| K00281 | GLDC, gcvP; glycine dehydrogenase [EC:1.4.4.2] | 0.036424 | 1.18E-07 | 0.410989 | 0.712535 | 0.038826 | 2.87E-07 | 0.604029 | 0.767954 | 0.528524 | -5.16333 | 0.125741 | 0.492411 |
| K00090 | ghrB; glyoxylate/hydroxypyruvate/2-ketogluconate reductase [EC:1.1.1.79 1.1.1.81 1.1.1.215] | 0.038905 | 1.47E-06 | 0.062858 | 0.003637 | 0.04102 | 2.46E-06 | 0.184765 | 0.023517 | 0.557145 | -4.06843 | -0.68973 | -1.7997 |
| K03840 | fldB; flavodoxin II | 0.040739 | 9.34E-07 | 0.045136 | 0.003637 | 0.042491 | 1.62E-06 | 0.150972 | 0.023517 | 0.577144 | -4.26586 | -0.83357 | -1.7997 |
| K00508 | E1.14.19.3; linoleoyl-CoA desaturase [EC:1.14.19.3] | 0.045051 | 0.680172 | 0.002675 | 0.605821 | 0.046489 | 0.680172 | 0.014415 | 0.667779 | 0.620837 | 1.596191 | -2.06078 | 0.421948 |
| K00130 | betB, gbsA; betaine-aldehyde dehydrogenase [EC:1.2.1.8] | 0.047454 | 4.06E-06 | 0.139799 | 0.038721 | 0.048453 | 5.55E-06 | 0.308193 | 0.101512 | 0.643408 | -3.62802 | -0.34259 | -0.77245 |
| K00311 | ETFDH; electron-transferring-flavoprotein dehydrogenase [EC:1.5.5.1] | 0.047974 | 0.001435 | 0.912048 | 0.121659 | 0.048474 | 0.001698 | 0.951276 | 0.23139 | 0.648142 | -1.07952 | 0.471928 | -0.27525 |
| K03807 | ampE; AmpE protein | 0.049212 | 2.41E-06 | 0.057049 | 0.003637 | 0.049212 | 3.54E-06 | 0.178508 | 0.023517 | 0.659206 | -3.85409 | -0.73184 | -1.7997 |
